# Case report: Primary intracranial mucosa-associated lymphoid tissue lymphoma presenting as two primary tumors involving the cavernous sinus and extra-axial dura, respectively

**DOI:** 10.3389/fonc.2022.927086

**Published:** 2023-01-04

**Authors:** Shiyun Tian, Tao Pan, Bingbing Gao, Wanyao Li, Jiashen Liu, Kun Zou, Yanwei Miao

**Affiliations:** ^1^ Department of Radiology, First Affiliated Hospital of Dalian Medical University, Dalian, China; ^2^ The Interventional Therapy Department, First Affiliated Hospital, Dalian Medical University, Dalian, China; ^3^ Department of Pathology, First Affiliated Hospital, Dalian Medical University, Dalian, China; ^4^ Department of Radiation Therapy, First Affiliated Hospital, Dalian Medical University, Dalian, China

**Keywords:** extranodal marginal zone lymphoma, mucosa-associated lymphoid tissue, primary intracranial tumor, central nervous system, magnetic resonance imaging, treatment

## Abstract

Primary intracranial mucosa-associated lymphoid tissue (MALT) lymphoma is a rare type of brain tumor, with only a few reported cases worldwide that mostly have only one lesion with conventional magnetic resonance imaging (MRI) findings. Here, we present a special case of intracranial MALT lymphoma with two mass lesions radiographically consistent with meningiomas on MRI before the operation. A 66-year-old woman was admitted to the hospital with intermittent right facial pain for 1 year, aggravated for the last month. Brain MRI showed two extracerebral solid masses with similar MR signal intensity. One mass was crescent-shaped beneath the skull, and the other was in the cavernous sinus area. Lesions showed isointensity on T1WI and T2WI and an intense homogeneous enhancement after contrast agent injection. Both lesions showed hyperintensity in amide proton transfer–weighted images. The two masses were all surgically resected. The postoperative pathology indicated extranodal marginal zone B-cell lymphoma of MALT. To improve awareness of intracranial MALT lymphoma in the differential diagnosis of extra-axial lesions among clinicians, we present this report and briefly summarize previously reported cases to describe the clinical, pathological, radiological, and treatment features.

## Introduction

Extranodal marginal zone lymphoma of the mucosa-associated lymphoid tissue (MALT lymphoma), as defined in the 2016 World Health Organization classification of lymphoid neoplasms (1), is a type of mature B-cell lymphoid neoplasm. At the very beginning, MALT lymphoma is described as a subtype of gastric lymphoma. Then, studies found that the MALT lymphoma may arise in almost all organs of the human body, including unusual sites, such as the dura (2). MALT lymphoma derived from dura is extremely rare; only a few cases were diagnosed and reported (3–10). The diagnoses are mostly based on conventional magnetic resonance imaging (MRI) image, and some combine with diffusion–weighted imaging (DWI) (11). This case report presented a MALT lymphoma with two separated lesions in the cranial: along the right fronto–temporo–parietal extra–axial dura and in the right cavernous sinus (CS). On the other hand, the patient also underwent amide proton transfer–weighted (APTw) and contrast–enhanced fluid–attenuated inversion recovery (FLAIR) examinations in addition to the conventional MR sequences.

## Case report

A 66–year–old woman was admitted to the hospital with intermittent right facial pain for 1 year, aggravated for the last month, the pain was knife–like accompanied with numbness of the upper and lower lips and had no obvious inducement before onset. The patient also had nausea without vomiting. No other objective neurologic findings were detected. The patient had a history of type 2 diabetes mellitus for more than 10 years.

**Figure 1:**
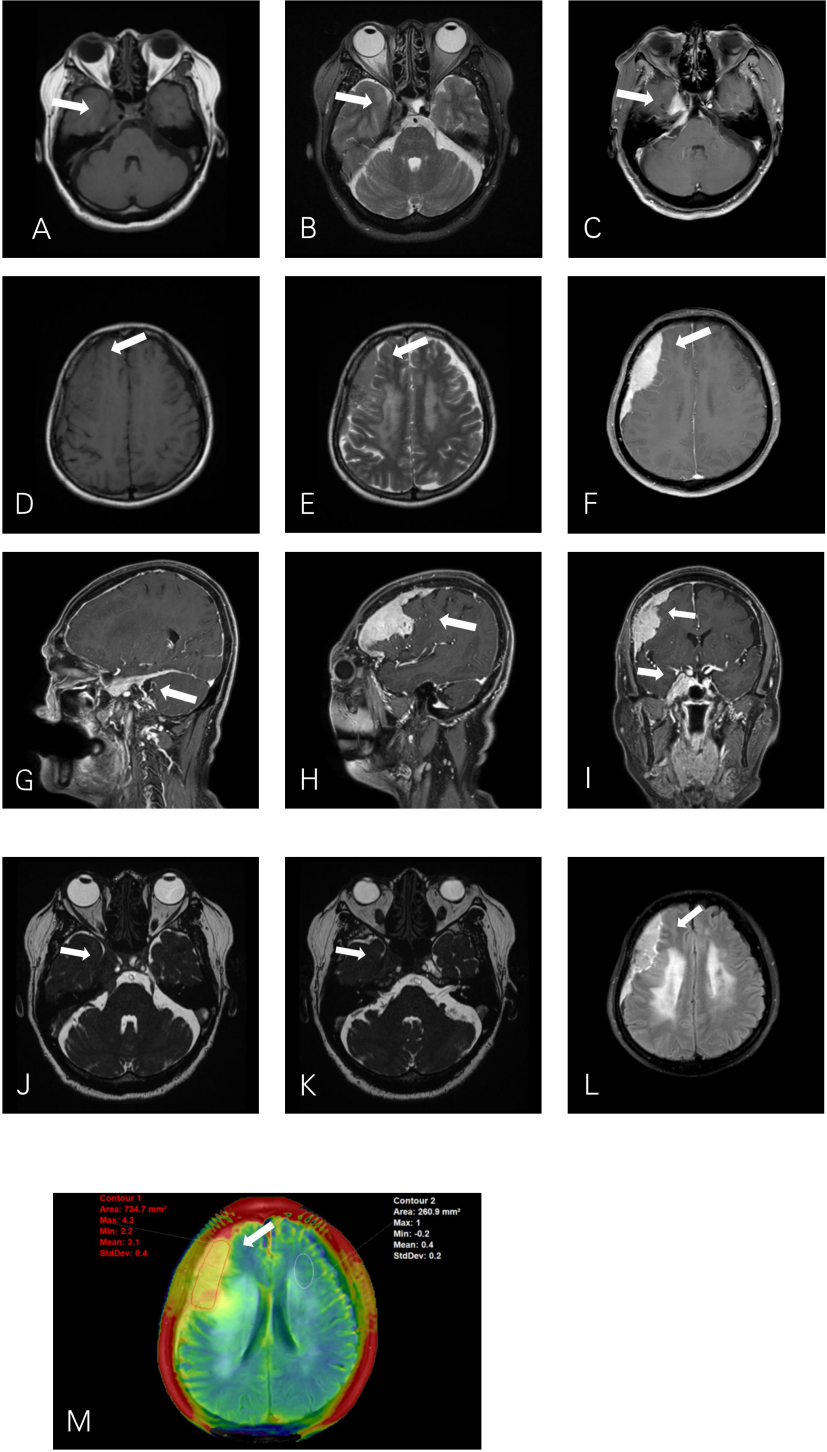
Axial T1WI **(A)** and T2WI **(B)** show a fusiform lesion isointensity to gray matter in the right cavernous sinus (CS). T1WI **(D)** and T2WI **(E)** show a crescent–shaped extra–axial mass in the right fronto–temporo–parietal region dura. The right CS lesion **(J)** extends into the posterior cranial fossa into the internal auditory canal and encircles the auditory nerve and facial nerve **(K)**. Post–contrast T1–weighted **(C, F, G, H, I)** images show intense homogeneous enhancement of the mass lesions with both dural tails visible. The leptomeningeal involvement of the right extra–axial dura lesion shows serrated enhancement on contrast–enhanced T2–FLAIR **(L)**. APTw image **(M)** shows a relative homogeneous tumor mass, with APTw signal intensity rates of 2.2%–4.3% (mean: 3.1%).

Brain MRI showed two extracerebral solid masses with similar MR signal intensity. One mass was fusiform in the cavernous sinus (CS) area (**Figures1 A–C**), the other was crescent-shaped beneath the fronto-temporo-parietal region skull (**Figures1 D–F**). The lesions showed a slight hypointensity to isointensity on T1–weighted image (T1WI), an isointensity on T2–weighted image (T2WI), and an intense homogeneous enhancement after contrast agent injection. (**Figures 1G–I**). For more details, for the tumor beneath cranium, short striped flow voids signals inside and moderate edema zone around (**Figure 1E**) are were observed. As the CS lesion, the ipsilateral cavernous segment of internal carotid artery had no stenosis (**Figures 1J, K**). The right CS lesion spread to the posterior cranial fossa, involving the internal auditory canal and encircled the cisternal segment of the right trigeminal nerve, auditory nerve and facial nerve (**Figures 1J, K**). The serrated enhancement was due to leptomeningeal involvement along the inner side of the fronto-temporo-parietal region lesion on the post-contrast fluid-attenuated inversion recovery (FLAIR) image (**Figure 1L**). APTw image (**Figure 1M**) showed relatively hyperintense compared to the normal brain tissue, the mean APTw value of the tumor was 3.1% (2.2%–4.3%). Overall, the MR features of these two lesions were misdiagnosed asconsistent with meningiomas.

The surgery was performed to resect the lesions on the right forehead and parasellar *via* the right frontotemporal parietal approach. The tumor was 9.0 × 7.5 cm in size at the top of the right forehead region, reaching from the base of the anterior cranial fossa forward to the central anterior gyrus backward. The solid tumor showed inconsistent hardness supplied by dural and lateral fissure vessels and adheres closely to the brain parenchyma. The dura near the tumor was enlarged up to 2 cm. The lesion was completely removed. The right parasellar tumor was relatively soft grayish white and dark brown mass with a size of 2.5 × 2.0 × 1.8 cm, wrapping the internal carotid artery and III–V cranial nerves. The parasellar tumor is only partially removed along the internal carotid artery and the lateral cranial nerve space due to the involvement of the cranial nerve.

**Figure 2:**
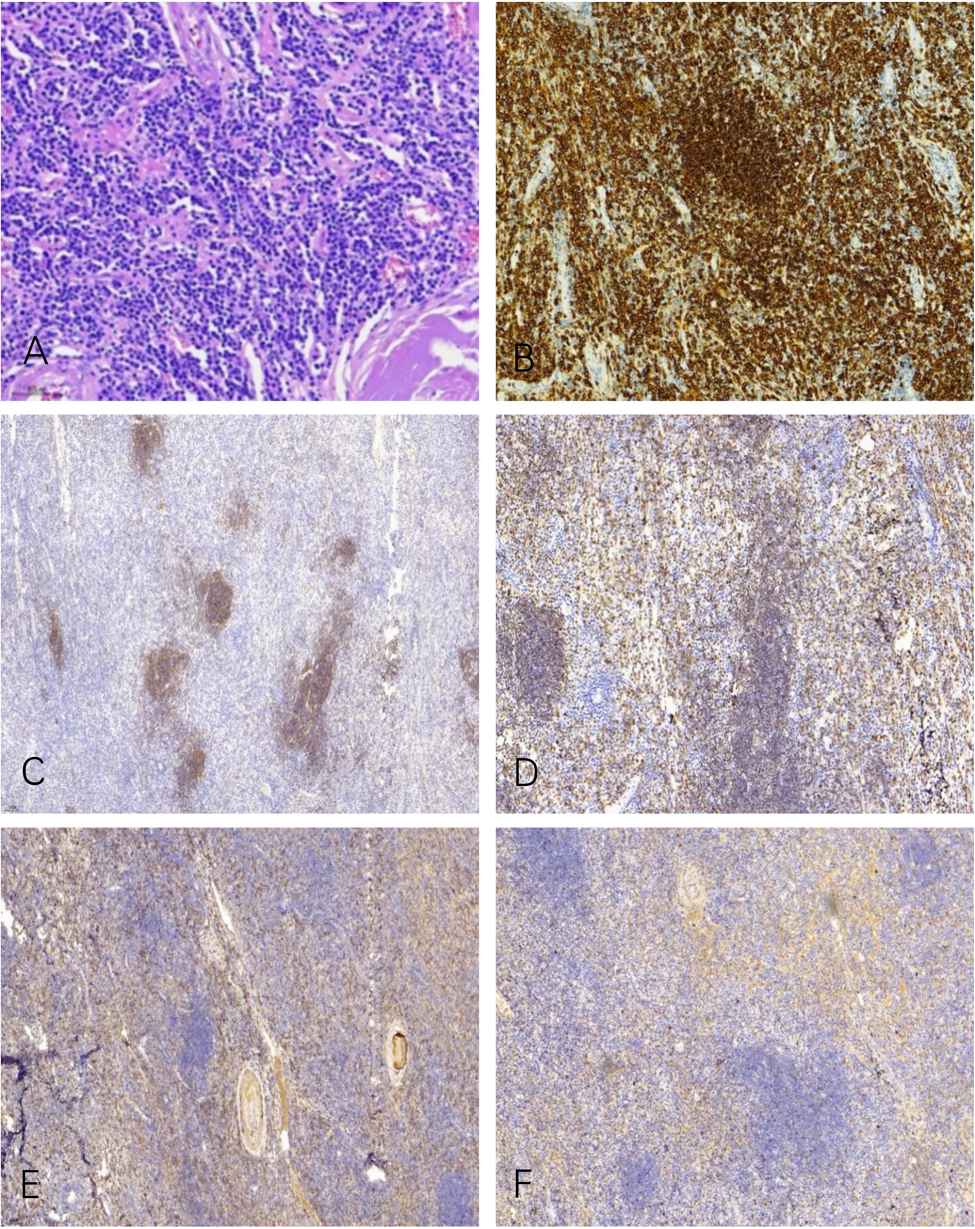
Microscopic examination shows a diffuse infiltrate of lymphocytes, plasma cells, and some cells with intermediate lymphoplasmacytic morphology **(A)**. The lymphocytic infiltrates were positive for CD20 **(B)**, CD21 **(C)**, and CD79a **(D)**. These lymphocytes and plasma cells are monotypic for kappa light chain **(E)** expression, and they are essentially negative for lambda light chain **(F)**.

Microscopic examination of the biopsy revealed fragments of dense connective tissue infiltrated by closely packed, medium–sized, and monomorphic lymphocytes and scattered plasma cells (**Figure 2A**). The lymphocytic infiltrates were positive for CD20 (**Figure 2B**), CD138, CD38, CD21 (**Figure 2C**), CD79a (**Figure 2D**), and MUM1. These lymphocytes and plasma cells are monotypic for kappa light chain (**Figure 2E**) expression, and they are essentially negative for lambda light chain (**Figure 2F**). The pathological diagnosis was MALT lymphoma with plasma cell differentiation and amyloidosis of the stroma and vascular wall.

**Figure 3:**
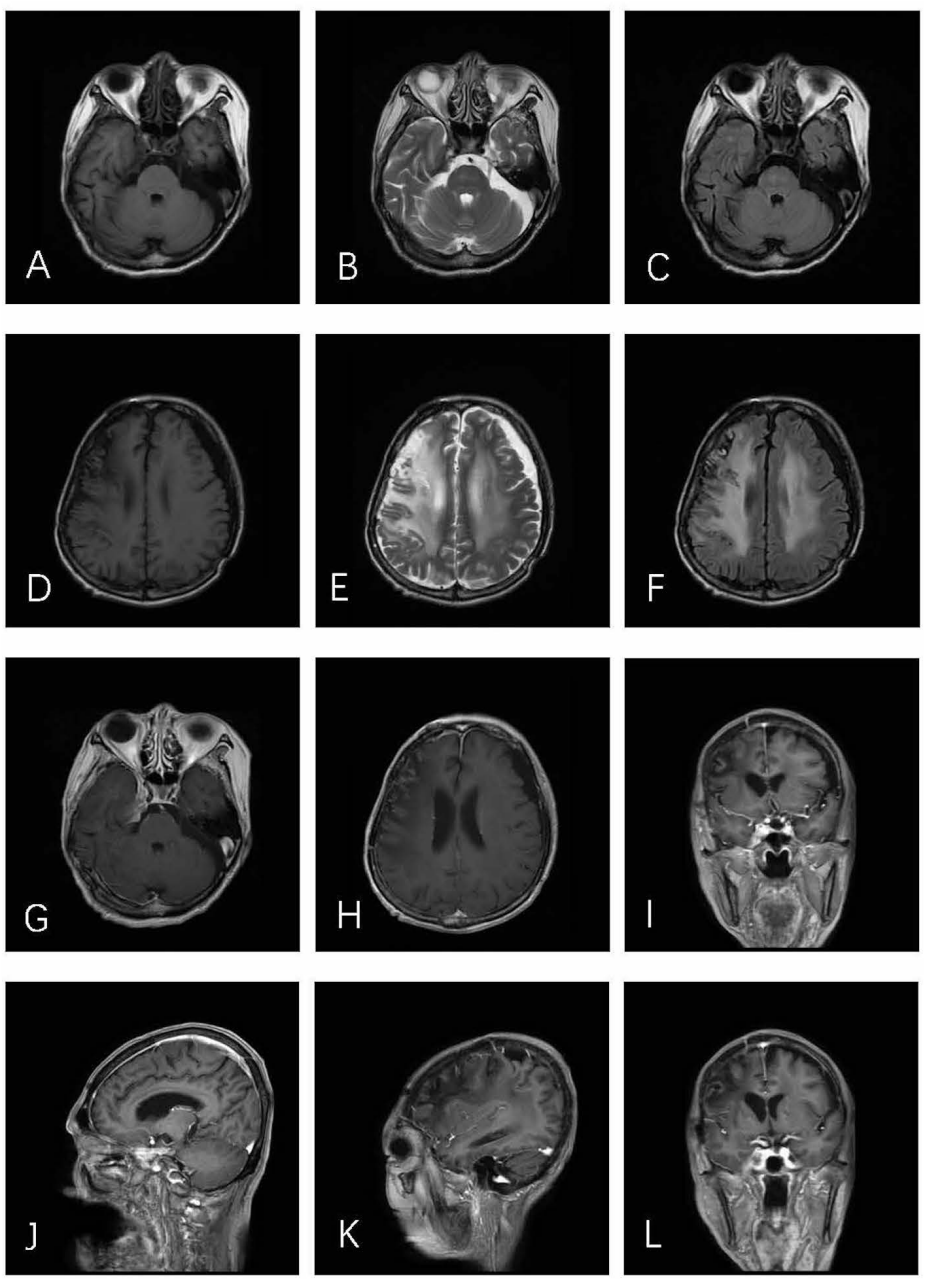
Axial T1WI **(A)**, T2WI **(B)**, and T2–FLAIR **(C)** demonstrate postoperative alterations in CS. In axial T1WI **(D)**, T2WI **(E)**, and T2–FLAIR **(F)** images, an obvious edema zone can be seen in the right parietal frontal brain, which may be related to brain changes after radiotherapy. No tumor recurrence was found in post–contrast T1–weighted **(G–L)** images.

A month and a half after the surgery, the patient received 4 weeks of whole–brain radiotherapy of 40 Gy in total. Follow–up of the initial MRI 8 months after the operation illustrated no evidence of tumor recurrence (**Figures 3A–L**). During the last 27 months of follow–up, the patient had no complaints in her daily life.

## Discussion

Extranodal marginal zone MALT lymphoma accounts for 7% to 8% of newly diagnosed lymphomas (12). Although the World Health Organization classification published in 2008 still defines the stomach as the most common organ of origin (accounting for roughly 50% of MALT lymphomas), recent data have suggested a decline in the percentage of gastric MALT lymphomas (12). It can occur in a variety of extranodal locations, but it is most common in organs where lymphocytes are generally absent, such as the stomach, salivary glands, and thyroid. To our knowledge, only a few cases have been reported to occur in the dura mater (3, 4). Louveau et al. (12) discovered functional lymphatic vessels in the dural sinus, which could be the cause of the issue. The published cases were all single lesions. To the best of our knowledge, we are the first to present a rare case of two simultaneous lesions occurring in different cranial regions.

MALT lymphoma has a wide range of clinical presentations, owing to variances in signs and symptoms associated with different extranodal organs. According to the literature, there is a slight female preponderance among patients with MALT lymphoma, and the median age at diagnosis is about 65 years. Patients with cranial MALT lymphoma present non–specific neurologic symptoms, including headache, meningeal signs, and cranial nerve involvement (4). The clinical features are summarized in **Table 1**. In this case, the patient complained about a severe knife–like pain on the right side of her face. The MRI showed that the trigeminal nerve was involved by the right CS lesion, and it was further confirmed that the lesion wrapped the III–V nerves during the operation.

**Table 1:** Summary of Intracranial MALT Lymphomas up to 4 November 2022.

References	Age/sex	Immune status	Clinical features	Involvement site	CT/MRI	Treatment	Follow–up
Shaia et al. (2)	61/F	N/A	Nausea and vomiting	Right posterior fossa	−/+	Oral steroid therapy, surgery, R/T	3/6 months: relapse–free
Ferguson et al. (4)	29/F	Immunocompetent	Exophthalmos and visual loss	Right optic foramen and cavernous sinus	−/+	Surgery, R/T	3 years: relapse–free
Choi et al. (5)	69/M	N/A	Headache	Anterosuperior	−/+	Surgery, C/T	30 months: relapse–free
Sanjeevi et al. (6)	46/F	N/A	Headache and ophthalmalgia	Left cavernous sinus	−/+	surgery, R/T	N/A
Neidert et al. (8)	44/M	N/A	facial numbness	right fronto–parietal	−/+	surgery, R/T	2 years: relapse–free
Yang et al. (11)	59/M	Immuno competent	Ptosis and blurred vision	Right cavernous sinus	+/+	Surgery, R/T, C/T	2 years: relapse–free

N/A, not available, C/T, chemotherapy, R/T, radiotherapy.

-/+ represents the absence or availability of corresponding CT and MRI images.

Radiologically, MALT lymphoma is mainly a single hyperattenuated lesion on CT, reflecting the highly dense tumor cells. MRI reveals a homogeneous isointensity–to–hypointensity lesion on T1WI and isointensity on T2WI, consistent with the dense cells and increased fibrous tissues in the tumor. Vasogenic edema is typically noted in the adjacent brain parenchyma. MALT lymphomas are uniformly enhanced on post–contrast T1WIs, and the interface between the tumor and brain parenchyma is blurred (13), with a long and wide meningeal tail sign. In this case, the contrasted T2–FLAIR sequence shows a sawtooth–like enhancement in the brain–tumor interface, which is considered as a sign of the involvement of the pia mater. The permeability of meningeal vessels increases with the breakdown of the blood–brain, blood– cerebrospinal fluid (CSF), or blood–nerve barrier (14). As T2–FLAIR sequences are thought not to show signals in the leptomeningeal vasculatures at a normal flow, a leptomeningeal enhancement on the contrast–enhanced T2–FLAIR images indicates a true leptomeningeal involvement (8, 15, 16).

Advanced MRI technology can add more diagnostic information and improve the accuracy of diagnosis. However, to our knowledge, only one MALT lymphoma case with DWI has been published. They observed diffusion restriction on DWI (b–value =1,000 s/mm^2^) with a decreased apparent diffusion coefficient (ADC) value of 0.64 × 10^−3^ mm^2^/s (11). The patient in this case underwent APTw sequence, and a relative hyperintense mass with a mean APTw value of 3.1% in the APTw. The elevated APTw may be result from a high protein and amino acid concentration in the increased lymphoid cells. The APTw changes are also helpful to the differentiation and grading of tumors. Jiang et al. (16) found that the APTW_max_ value of lymphomas was lower than that of high–grade gliomas (3.38% ± 1.06% and 4.36% ± 1.30%, respectively). Recent studies on meningiomas have demonstrated that the normalized magnetization transfer ratio asymmetry (nMTR_asym_) of atypical meningiomas was significantly greater than that of benign meningiomas (2.46% *vs*. 1.67%, P < 0.001) (17).

A wide variety of lesions can involve the dura, ranging from benign to malignant neoplastic, infectious, and granulomatous etiologies. Frequently, these lesions have imaging characteristics similar to those of a meningioma and are often mistaken for one. MALT lymphoma is often misdiagnosed as meningioma because of its origin in the dura mater. As shown in this case, compared with meningiomas, the meningeal tail of MALT lymphoma is longer and not smooth, and the interface presents a serrated change. Solitary fibrous tumor/hemangiopericytomas also originate from dura and is characterized by a narrow base connected to the dura, and “mushroom” grows to the adjacent brain in a lobulated shape. Intracranial Rosai–Dorfman disease is a rare benign histiocytosis that primarily affects men. The distinguishing point from MALT lymphoma is that the edema of the adjacent brain parenchyma is obvious.

The treatment of MALT lymphoma includes local surgical resection, radiotherapy, or chemotherapy. This case was misdiagnosed as meningiomas and underwent surgical resection. During the operation, the tumor adhered to the cranial nerve, resulting in a small part of the residual. Radiotherapy was performed after the operation. The patient’s life was normal after postoperative radiotherapy. Although surgical resection of tumors may cure many patients with MALT lymphoma, the use of this strategy is gradually decreasing (12). This is because postoperative sequelae and organ dysfunction are more harmful than lymphoma itself (12). In one case (3), six cycles of rituximab + bendamustine allowed intracranial MALT lymphoma complete remission for more than 2 years without the need for invasive surgery. Therefore, surgery is mainly limited to histopathological diagnosis, management of treatment complications, or treatment of recurrent diseases in patients who are not suitable for other treatments. As an inert malignant tumor, MALT lymphoma usually has a good curative effect.

In conclusion, we report a case in which two MALT lymphomas occur simultaneously in different skull regions. These specific MRI features combined with APTw, post–contrast T1WI, and contrast–enhanced T2 FLAIR images could help in making a directive diagnosis before the operation, which can further help neurosurgeons make appropriate preoperative treatment plans for patients.

## Data availability statement

The original contributions presented in the study are included in the article/supplementary material. Further inquiries can be directed to the corresponding author.

## Ethics statement

The studies involving human participants were reviewed and approved by the Local Ethics Committee of the First Affiliated Hospital of Dalian Medical University. The patients/participants provided their written informed consent to participate in this study. Written informed consent was obtained from the individual(s) for the publication of any potentially identifiable images or data included in this article.

## Author contributions

ST and TP reviewed the literature, designed the article and wrote the report. BG and WL collected and analyzed the data. JL prepared histology figures and provided immunohistochemical analysis. KZ provided information on radiotherapy. YM revised the report critically for important intellectual content and gave final approval of the version to be published. All authors contributed to the article and approved the submitted version.
